# Drug resistance associated genetic polymorphisms in *Plasmodium falciparum *and *Plasmodium vivax *collected in Honduras, Central America

**DOI:** 10.1186/1475-2875-10-376

**Published:** 2011-12-19

**Authors:** Irina T Jovel, Rosa E Mejía, Engels Banegas, Rita Piedade, Jackeline Alger, Gustavo Fontecha, Pedro E Ferreira, Maria I Veiga, Irma G Enamorado, Anders Bjorkman, Johan Ursing

**Affiliations:** 1 Malaria Research Laboratory, Infectious Diseases Unit, Department of Medicine, Karolinska University Hospital/Karolinska Institutet, Retzius väg 10, 171 77 Stockholm, Sweden; 2 Departamento de Parasitología, Escuela de Microbiología, Facultad de Ciencias, Universidad Nacional Autónoma de Honduras (UNAH), Tegucigalpa, Honduras; 3 Laboratorio Nacional de Malaria, Departamento de Laboratorios, Secretaría de Salud, Tegucigalpa, Honduras; 4 Unit of Drug Resistance, Division of Pharmacogenetics, Department of Physiology and Pharmacology, Karolinska Institute, Stockholm, Sweden; 5 Institute of Biotechnology and Bioengineering, Centre of Molecular and Structural Biomedicine, University of Algarve, Faro, Portugal; 6 Servicio Parasitología, Departamento de Laboratorios Clínicos, Hospital Escuela; Unidad de Investigación Científica, Facultad de Ciencias Médicas, Universidad Nacional Autónoma de Honduras, Tegucigalpa, Honduras; 7 Maestría de Enfermedades Infecciosas y Zoonóticas (MEIZ), Escuela de Microbiología, Facultad de Ciencias, Universidad Nacional Autónoma de Honduras (UNAH), Tegucigalpa, Honduras; 8 Department of Microbiology, Tumor and Cell Biology (MTC), Karolinska Institutet, Stockholm, Sweden; 9 Departamento de Bioanálisis e Inmunología, Escuela de Microbiología, Facultad de Ciencias, Universidad Nacional Autónoma de Honduras (UNAH), Tegucigalpa, Honduras

**Keywords:** *Plasmodium falciparum*, *Plasmodium vivax*, Chloroquine, Sulphadoxine-pyrimethamine, Single nucleotide polymorphisms and drug resistance

## Abstract

**Background:**

In Honduras, chloroquine and primaquine are recommended and still appear to be effective for treatment of *Plasmodium falciparum *and *Plasmodium vivax *malaria. The aim of this study was to determine the proportion of resistance associated genetic polymorphisms in *P. falciparum *and *P. vivax *collected in Honduras.

**Methods:**

Blood samples were collected from patients seeking medical attention at the Hospital Escuela in Tegucigalpa from 2004 to 2006 as well as three regional hospitals, two health centres and one regional laboratory during 2009. Single nucleotide polymorphisms in *P. falciparum *chloroquine resistance transporter (*pfcrt*), multidrug resistance 1 (*pfmdr1*), dihydrofolate reductase (*pfdhfr*) and dihydropteroate synthase (*pfdhps*) genes and in *P. vivax *multidrug resistance 1 (*pvmdr1*) and dihydrofolate reductase (*pvdhfr*) genes were detected using PCR based methods.

**Results:**

Thirty seven *P. falciparum *and 64 *P. vivax *samples were collected. All *P. falciparum *infections acquired in Honduras carried *pfcrt*, *pfmdr1, pfdhps *and *pfdhfr *alleles associated with chloroquine, amodiaquine and sulphadoxine-pyrimethamine sensitivity only. One patient with parasites acquired on a Pacific Island had *pfcrt *76 T and *pfmdr1 *86Y alleles. That patient and a patient infected in West Africa had *pfdhfr *51I, 59 R and 108 N alleles. *Pvmdr1 *976 F was found in 7/37 and two copies of *pvmdr1 *were found in 1/37 samples. *Pvdhfr *57 L + 58 R was observed in 2/57 samples.

**Conclusion:**

The results indicate that *P. falciparum *from Honduras remain sensitive to chloroquine and sulphadoxine-pyrimethamine. This suggests that chloroquine and sulphadoxine-pyrimethamine should be efficacious for treatment of uncomplicated *P. falciparum *malaria, supporting current national treatment guidelines. However, genetic polymorphisms associated with chloroquine and sulphadoxine-pyrimethamine tolerance were detected in local *P. vivax *and imported *P. falciparum *infections. Continuous monitoring of the prevalence of drug resistant/tolerant *P. falciparum *and *P. vivax *is therefore essential also in Honduras.

## Background

In Honduras chloroquine is recommended for treatment of uncomplicated *Plasmodium falciparum *and *Plasmodium vivax *infection. In addition primaquine is used for treatment of *P. falciparum *gametocytes and *P. vivax *hypnozoites [1,2]. These drugs are believed to remain effective despite being used for six decades and despite the spread of chloroquine resistance across most of the rest of the world. However, there is lack of clinical trials as well as *in vitro *studies and the proportions of resistance associated genetic polymorphisms have not been determined in Honduras or other Mesoamerican countries [3].

There are several genetic polymorphisms described in *P. falciparum *and *P. vivax *that can provide reliable data about the prevalence of drug resistance. The most relevant polymorphisms are presented below. The 76 T allele in the chloroquine resistance transporter gene (*pfcrt*) is predictive of chloroquine and amodiaquine treatment failure [4-6]. The 86Y allele of the multidrug resistance gene 1 (*pfmdr1*) has been linked with chloroquine and amodiaquine resistance and increased chloroquine inhibitory concentrations in *P. falciparum *with *pfcrt *76 T [7]. The *pfcrt *76 K and *pfmdr1 *86 N alleles have been associated with lumefantrine tolerance and higher lumefantrine IC50 values [8-10]. Amplifications of *pfmdr1 *have been associated with mefloquine resistance, lumefantrine tolerance and reduced sensitivity to artesunate [11-13]. The triple dihydrofolate reductase (*pfdhfr*) haplotype N51I/C59R/S108N has been associated with sulphadoxine-pyrimethamine (SP) treatment failure and when dihydropteroate synthase (*pfdhps*) SNPs G437A and K540E are added, highly resistant *P. falciparum *are generated [14-18].

In *P. vivax*, the multidrug resistance gene 1 (*pvmdr1*) 976 F allele has been associated with reduced susceptibility to chloroquine and increased susceptibility to mefloquine and artesunate. However, amplifications of *pvmdr1 *have been associated with reduced susceptibility to mefloquine and artesunate [18-20]. Twenty single nucleotide polymorphisms have been described in *P. vivax *dihydrofolate reductase (*pvdhfr*) including F57L, S58R, T61M and S117N/T that correspond to codons 50, 51, 59 and 108 in *pfdhfr*, respectively [21-24]. *Pvdhfr *S58R and S117N result in decreased binding of pyrimethamine [25] and quadruple (F57L, S58R, S117N and I173L) resistance associated SNPs have been associated with SP treatment failure [21-23].

The aim of the study was to determine the proportion of resistance associated genetic polymorphisms in *P. falciparum *and *P. vivax *field samples collected in Honduras.

## Methods

### Study setting, participants and ethics

**Figure 1:**
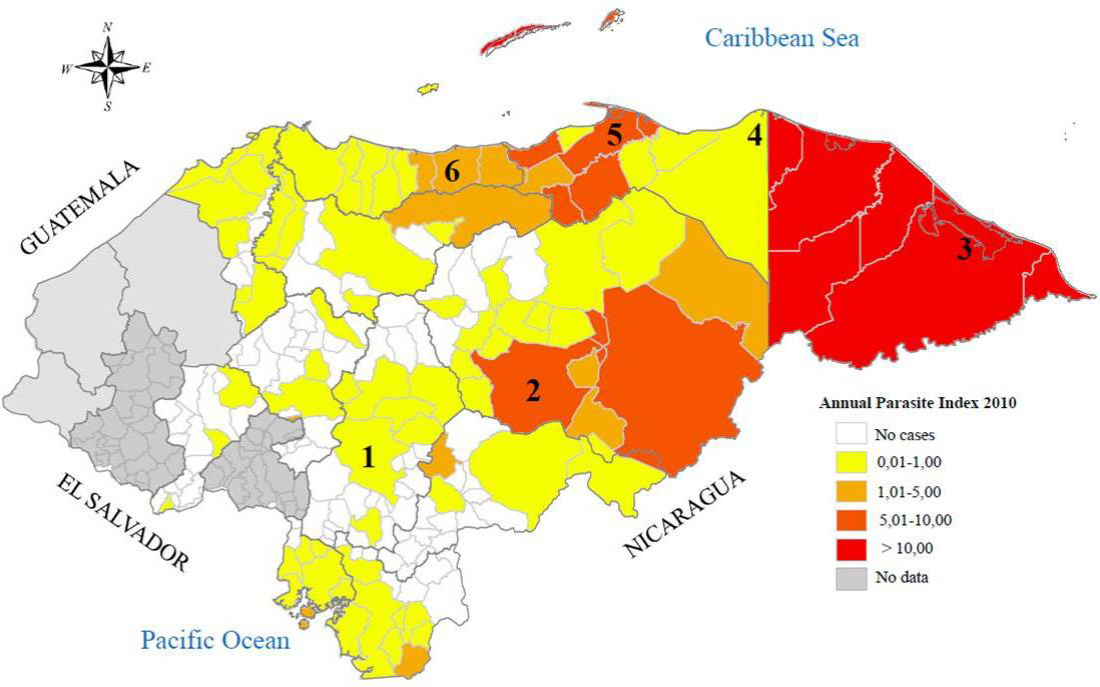
**Distribution of malaria in the 128 municipalities in Honduras**. Annual Parasite Incidence per 1,000 habitants. Samples were collected in (1) Distrito Central-Tegucigalpa, (2) Juticalpa, (3) Puerto Lempira, (4) Iriona, (5) Trujillo, (6) La Ceiba. The figure has been modified from [2] to provide more detail.

In Honduras malaria transmission is seasonal and in 2010 there were 9,078 reported malaria cases. *Plasmodium vivax *mono-infection accounted for 88% and 12% were due to *Plasmodium falciparum *mono-infection and mixed *P. vivax *and *P. falciparum *infections. The country is divided into 20 health regions and there are 1,743 health facilities [2]. Samples were collected from patients that sought medical attention at the Hospital Escuela that is a teaching hospital in Distrito Central-Tegucigalpa, the regional hospitals in Trujillo, La Ceiba and Juticalpa, two primary health centres in Puerto Lempira and Iriona and one regional laboratory in Juticalpa. Samples were collected at Hospital Escuela between 2004 and 2006 and during 2009 at all other sites. The locations of all sites are shown in Figure 1. At the Hospital Escuela sample collection was considered to be part of routine malaria surveillance and did not involve additional sampling or collection of patient data. At the other health facilities patients who sought medical attention and were diagnosed with malaria were invited to participate in the study after written informed consent. The study was approved by the ethical review committee of Cardio Pulmonary National Institute in Tegucigalpa, Honduras. The molecular analyses were approved by the Stockholm regional ethics board (reference number 2011/832-32/2).

### Sample collection and microscopy

As part of routine practice, 3 ml of blood were collected in EDTA tubes by venepuncture at the Hospital Escuela. Thick and thin smears were made and stained using Giemsa. Slides were viewed under X100 magnification. Trained laboratory technician examined at least 100 microscopy fields before considering a sample negative. When *P. falciparum *or *P. vivax *were identified approximately 100 μL of blood was collected on a filter paper (Schleicher & Schuell 903) that was dried, placed inside a small plastic bag and stored at room temperature. At the other health units the samples were generally collected in the same way but occasionally capillary samples were obtained by finger puncture. Microscopy was quality controlled at the National Malaria Laboratory were microscopists routinely re-examine all positive and approximately 10% of negative slides.

### Sample storage, DNA extraction and amplification

DNA was extracted from the filter papers using an ABI Prism^® ^6100 Nucleic Acid Prep Station (Applied Biosystems, Fresno, CA) according to the manufacturer's instructions with minor modifications [26]. Extracted DNA was stored at-20°C.

Previously described multiplex PCR-RFLP (restriction fragment length polymorphism) methods were used to identify the following SNPs; *pfcrt *K76T, *pfmdr1 *N86Y, *pfdhfr *N51I, C59R, N108T/S and *pfdhps *G437A and K540E [27]. *Pfcrt *72-76 haplotypes were identified by PCR amplification followed by sequencing [28]. Previously described nested PCRs were used to amplify codons 917-1118 of *pvmdr1 *and codons 1-238 of *pvdhfr *[19,29]. *Pvmdr1 *and *pvdhfr *SNPs were then identified by sequencing. The Sequencher ™ software version 4.6 (Gene Codes Corporation, Ann Arbor, MI) was used for sequencing analysis. The *P. falciparum *3D7 clone sequence obtained from NCBI database (*pfcrt *Gen-Bank Accession no. NC_004328) was used as reference for *pfcrt*. For *pvdhfr *the *P. vivax *ARI/Pakistan isolate sequence (Gen-Bank accession no. X98123) and for *pvmdr1 *the *P. vivax *Sal-1 isolate sequence (Gen-Bank accession no. AY618622) were used as references. PCR and restriction products were resolved on 2% agarose gels (Amresco, Solon, OH). All gels were stained with ethidium bromide and visualized under UV transillumination (GelDoc^®^, Biorad, Hercules, Ca, USA). PCR products were purified and sequenced commercially (Macrogen Inc. Seoul, Korea).

*Pfmdr1 *and *pvmdr1 *copy numbers were determined using real time PCR (ABI Prism^® ^7000 Sequence Detection System) as previously described [12,30]. All samples were run in triplicate. For *pfmdr1 *3D7, D10 and K1 clones were used as single copy calibrators and FCB and Dd2 were multiple copy controls. *Pvmdr1 *single and double copy calibrators were created by the insertion of *pvmdr1 *nucleotides 2751-3354 and *pvbtubulin *nucleotides 860-1056 in the pCR2.1 vector using the TOPO TA-cloning kit (Invitrogen, Carlsbad, CA) at 1:1 and 2:1 proportions, respectively. The sample copy numbers were calculated using a comparative threshold method (ΔΔC_t_). Copy number > 1.6 was defined as a duplication of the aforementioned genes. Assays were repeated if the following results were obtained: copy number 1.3-1.6 or Ct value > 35 or standard deviation value > 0.5.

### Power calculation and statistical analyses

This was an exploratory study and a power calculation of sample size was therefore not done. Data were entered, validated and analysed on Microsoft Excel 2003. For both species allele proportions were calculated by dividing the number of samples with a certain allele by the number of samples with an identifiable allele at that position. Thus mixed infections contributed to the proportion of both alleles.

## Results

### Sample collection

At the Hospital Escuela blood samples were collected from 29 patients with *Plasmodium falciparum *mono-infection, 15 patients with *Plasmodium vivax *mono-infection and four patients with mixed infections. Two patients with *P. falciparum *mono-infection were assumed to have contracted their infections on a Pacific island and in West Africa based on their respective travel history. At the other laboratories and health centres samples were collected from four patients with *P. falciparum *and 45 patients with *P. vivax *mono-infections. Further details are shown in Table 1.

**Table 1:** Origin of samples

Municipalities	*P. falciparum*	*P. vivax*	Mix inf
**Distrito Central-**	29	15	4
**Tegucigalpa**			
**Juticalpa**		26	
**Puerto Lempira**	2	3	
**Iriona**	2		
**Trujillo**		8	
**La Ceiba**		8	

*Two patients contracted malaria elsewhere: A tourist who was admitted one week after arriving in Honduras from the Pacific island region where he had been travelling. A pilot who was admitted the day he came to Honduras after working and visiting Equatorial Guinea, Ghana and Cameron during the previous three months.

### Polymorphisms in Plasmodium falciparum

*Pfcrt *K76T, *pfmdr1 *N86Y, *pfmdr1 *copy numbers, *pfdhfr *SNPs and *pfdhps *SNPs were successfully identified in 32/37 (91%) of samples. Allele proportions are presented in Table 2. All *P. falciparum *from Honduras (n = 30) had the *pfcrt *72-76 CVMNK haplotype. The patient that became infected with *P. falciparum *in West Africa had the *pfcrt *72-76 CVMNK haplotype and the patient infected on a Pacific island had the SVMNT haplotype. No other *pfcrt *SNPs were found in any of the sequenced fragments. Both imported cases had the *pfmdr1 *86Y allele and *pfdhfr *triple mutation 51I + 59 R + 108 N. One *pfmdr1 *copy was found in all samples.

**Table 2:** Single nucleotide polymorphisms and amplifications in *pfcrt, pfmdr1*, *pfdhfr *and *pfdhps*

Origin	*pfcrt*	*pfmdr1*		*Pfdhfr*			*Pfdhps*		Number (Proportion %)
	76	86	Copy Number	51	59	108	437	540	
**Honduras**	K	N	1	N	C	S	A	K	30 (94)^§^
**Pacific**	**T**	**Y**	1	**I**	**R**	**N**	A	K	1 (3)
**Africa**	**K**	**Y**	1	**I**	**R**	**N**	**G**/A	K	1 (3)

*Resistance associated alleles are shown in bold

^§^All infections were acquired in Honduras

### Polymorphisms in Plasmodium vivax

PCR amplifications of *pvmdr1 *were successful in 64/64 samples. Following sequencing, codon 976 was identified in 41 samples and codon 1076 in 37 samples. Proportions of the alleles are shown in Table 3. The double mutation 976 F + 1076 L was observed in 4/37 (11%) samples. No other *pvmdr1 *SNPs were found in the sequenced fragments. *Pvmdr1 *gene copy numbers were successfully quantified in 61/64 samples. Two *pvmdr1 *gene copies were found in one sample and one sample repeatedly had an indeterminate 1.5 copies. These results were verified by performing the real time PCR 5 times for these samples. The *pvmdr1 *gene amplification was found in *P. vivax *that had the 976Y.

**Table 3:** *Pvmdr1 *Y976F and F1076L haplotype proportions

Haplotypes	Y976F	F1076L	Number (Proportion %)
**1**	Y	F	29 (71)
**2**	Y	L	1 (2)
**3**	**F**	F	3 (7)
**4**	**F**	L	4 (10)
**5**	Y	ND	4 (10)

*Resistance associated alleles are shown in bold

*ND*: Not Determined

*Pvdhfr *was successfully amplified in 59/64 (92%) samples and sequencing was successful in 57 samples. Allele Proportions are presented in Table 4. The 57 L + 58 R double mutation was seen in 2/57 (3%) samples. Synonymous SNPs in codon 69 (TAT → TAC) were found in 13/57 (22%) samples. Two novel SNPs in codons 39 (CTG → TTG) and 85 (TCA → TCC) were observed in 2 (3%) and 3 (5%) samples, respectively. A repetition (ACA AGC GGT GGT GAC AAC) between amino acids 103 and 104 of *pvdhfr *was found in 5/57 (9%) of the samples.

**Table 4:** *Pvdhfr *F57L, S58R, T61M and S117N/T haplotype proportions

Alleles	F57L	S58R	T61M	S117N/T	Number (Proportion %)
**1**	F	S	T	S	57 (97)
**2**	**L**	**R**	T	S	2 (3)

*Resistance associated alleles are shown in bold

## Discussion

This is the first report on molecular markers of drug resistance from Honduras and indeed from Mesoamerica [3]. Finding no SNPs associated with CQ or SP resistance in *Plasmodium falciparum *from Honduras indicates that *P. falciparum *of Honduran origin remain CQ and SP sensitive. The results are in line with the continued efficacy of CQ seen in previous *in vitro *(1980) and *in vivo *(1980 and 1998-2000) studies [31,32]. This study thus indicates that the use of CQ in combination with primaquine for treatment of uncomplicated *P. falciparum *malaria and SP when CQ fails as recommended in the national treatment guidelines should be efficacious [1]. However, 2/2 patients with imported malaria had ≥ 3 SNPs associated with SP resistance suggesting that SP is not optimal for treating infections from regions outside Mesoamerica as is also recommended in the national treatment guidelines.

The results indicate that *pfcrt *76 K, *pfmdr1 *86 N and SNPs in *pfdhfr *and *pfdhps *associated with SP sensitivity are fixed in Honduras. This suggests that *P. falciparum *in Honduras are less susceptible to lumefantrine and possibly mefloquine compared to areas where the dominant SNPs are *pfcrt *76 T and *pfmdr1 *86Y [8-10,33]. Should Honduras wish to introduce an artemisinin-based treatment, combinations containing amodiaquine or SP might, from the point of view of drug sensitivity, be preferred over combinations containing lumefantrine or mefloquine.

The high proportion of patients with *P. falciparum *compared to *Plasmodium vivax *infection at the Hospital Escuela was most probably due patients with *P. falciparum *being referred there. The two patients with imported malaria had according to their history of travel contracted *P. falciparum *on a Pacific island and in West Africa. Finding the *pfcrt *72-76 SVMNT and CVMNK haplotypes is consistent with their respective travel histories suggesting that the resistance associated SNPs found were truly imported [34,35]. These two patients highlight the risk of importing drug resistant malaria to Honduras. Considering this it is worth noting that malaria is often diagnosed clinically in Honduras and that it is then treated presumptively with primaquine (0,25 mg/kg) for 5 days in addition to CQ. Primaquine reduces the gametocytes carriage time thus reducing transmission and it is certainly possible that this has contributed to stop resistant *P. falciparum *from becoming established [36,37].

CQ resistant *P. vivax *has been reported from several Asian and South American countries including Brazil, but not French Guiana [38]. In Southeast Asia the *pvmdr1 *976 F allele has been associated with reduced susceptibility to CQ [19] and in Brazil it was found in 100% of samples (n = 28) whilst it was not found in French Guiana (n = 5) [39,40]. Finding the *pvmdr1 *976 F allele in 7/41 (17%) *P. vivax *might thus indicate a degree of CQ tolerance but probably not resistance in Honduras [18,19,41]. In line with this, an *in vivo *evaluation conducted in Honduras between 1998 and 2000 found that 73/73 *P. vivax *infections were successfully treated with CQ and primaquine [32]. Finding two copies of *pvmdr1 *in 1/61 samples should be interpreted with caution as neither mefloquine nor artesunate are commonly used in Honduras [20]. It may however suggest the natural occurrence of this genetic change in Honduras. The prevalence of *pvmdr1 *1076 L varies considerably across the world and although an association with CQ resistance has been proposed none has yet been found [18,19,39-42]. The proportion in Honduras (4/37) is similar to that found in Brazil (4/28). In the *pvmdr1 *fragment amplified for this study one synonymous and nine non-synonymous SNPs have been reported worldwide. Five of these SNPs have been reported from South America in studies including nine patients whereas only one was found in this study [43,44].

Double (57 L + 117 N), triple (57 L + 58R + 117 N) and quadruple (57 L + 58R + 61 M + 117 T) *pvdhfr *mutations have been associated with SP resistant *P. vivax *[21,24,34]. In this study 2/57 samples had the double 57 L + 58 R SNPs that have previously been described in similar proportions in Asia but not in South America [21,24,29,45-48]. These SNPs may suggest a degree of tolerance but probably not resistance to SP. It is thus probable that SP should be efficacious for the treatment of *P. vivax *in Honduras [49]. However, the efficacy of SP for the treatment of *P. vivax *has not been assessed in Mesoamerica [3]. Furthermore, finding the double mutation despite a probably low consumption of SP suggests that resistance might develop rapidly if SP usage increases. A possible explanation for the occurrence of 57 L and 58 R despite the low use of SP for malaria might be that trimethoprim/sulfamethoxazole is the first line drug for treatment of acute respiratory tract infections in Honduras [50]. Two novel synonymous SNPs at codons 39 and 85, a previously described synonymous SNP at codon 69 and an insertion, were also found in *pvdhfr*. The proportion of SNP 69 (13/57) found in this study was considerably lower than that (77/90) reported from French Guiana [44].

## Conclusion

Only SNPs linked to CQ or SP sensitivity were found in *Plasmodium falciparum *originating in Honduras indicating that CQ and SP should remain efficacious. Genetic polymorphisms associated with CQ and SP tolerance were found in eight (13%) and two (3%) *Plasmodium vivax *samples, respectively, suggesting a degree of tolerance. CQ and SP resistance associated SNPs were found in patients that contracted *P. falciparum *overseas highlighting the risk of importing drug resistance to Honduras. Continuous monitoring of the prevalence of drug resistant/tolerant *P. falciparum *and *P. vivax *in Honduras is, therefore, essential.

## Competing interests

The authors declare that they have no competing interests.

## Authors' contributions

ITJ conducted molecular work, planned and wrote the first draft of the manuscript. REM, IGE, AB and JU planned the study and participated in writing the manuscript. EB and GF were responsible for sample collection from regional hospitals and health centres and participated in writing the manuscript. JA was responsible for sample collection from the Hospital Escuela and participated in writing the manuscript. RP, PF and MIV conducted molecular work and participated in writing the manuscript. All authors read and approved the final manuscript.
